# The Etiology of Periodontoclasia (Pyorrhea Alveolaris)

**Published:** 1920-04

**Authors:** Arthur H. Merritt

**Affiliations:** New York


### THE ETIOLOGY OF PERIODONTOCLASIA (PYORRHEA ALVEOLARIS)

ARTHUR H. MERRITT, D.D.S., NEW YORK

The etiology of periodontoclasia has been obscured by a number of things, among them being the complexity of the factors that act as causative agents, the variety of forms or stages in which the disease manifests itself, and in confounding it with other lesions which may in some respects simulate it, but which are not typical of periodontoclasia. It is further obscured by the fact that no two cases seem to be identical in their etiology or pathology. In one, the teeth are irregular and the mouth unclean with slight and more or less uniform shrinkage of the gums. In another, the teeth may be straight, the mouth clean and the pockets deep, with little or no gum recession. All of which has served to make "confusion worse confounded." When, however, one studies more closely these complex etiological factors, it will be found that they fall more or less naturally into two classes, which for convenience sake may be called primary and secondary. Again, a careful study of those causes arbitrarily placed in the primary class, will reveal the fact that irritation of some form, usually of a mechanical nature, is one of the most potent factors in the etiology of periodontoclasia. This irritation may express itself in two ways: irritation beginning at, and at first confined to the gingiva, and, secondly, irritation of such a nature as to affect the entire periodontal tissues—that is, the cementum, the pericementum and the alveolar process.

### GINGIVAL IRRITATION

The first of these, gingival irritation, is the more common, and as a rule the first to manifest itself. Anything which will chronically irritate the gingiva, no matter what it may be, must be regarded as a potential cause of periodontoclasia. This does not mean that every case of gingival irritation will eventuate in periodontoclasia, but it does mean that irritation of this tissue is potentially dangerous, and that the only insurance against possibly disastrous results is its maintenance in health. The gingiva spring from the crest of the alveolar process, and completely surround the cervical enamel. Between the free gingiva and the tooth is the subgingival space, the floor of which is bounded by the gingival group of pericemental fibers and is the point at which the first lesion occurs as a result of gingival irritation. The gingiva perform two very important functions, both of which are essential to the preservation in health of the teeth and their investing tissues. The first of these is the protection against injury of the attachment at the cemento-enamel junction. It is primarily a protective organ. In health and when of normal form, the free gingiva project themselves several millimeters above the alveolar crest, closely hugging the cervical enamel in such a way as to completely fill every interstice between the teeth, and terminating in a thin dense edge of such a shape as to shed off and prevent food lodgment. In childhood and in youth, when resistance is high, the gingiva will withstand an immense amount of abuse, but long continued neglect will eventually break down this protective barrier, thereby opening the door to some of the most serious diseases occurring in the mouth. The second function of the gingiva, and one closely related to the first in its protective character, is the maintenance of the teeth in their normal position in the dental arch. Their rigidity in their alveoli and their normal relation to each other and to the teeth in the opposing jaw, is due more to the healthy gingiva than to the alveolar process. The importance of this should not be overlooked in the study of occlusal trauma, inasmuch as it raises the question whether or not the latter may not be dependent for the exercise of its destructive influence upon a previous disturbance of the gingiva. It is probable that there is a closer relationship between diseases of the gingiva and traumatic occlusion than is generally realized. It is quite possible that the former antedates and may invite the latter.

Among those irritants which begin at and are at first confined to the gingiva, the most common, and the chief of offenders, is uncleanliness of the mouth. The habitual accumulation of food debris in, between and about the teeth, and especially in the subgingival space, together with the deposits associated with such neglect, are the chief sources of gingival irritation, and in turn responsible for more cases of periodontoclasia than all other causes combined. The importance of mouth cleanliness in its relation to this disease can not be overestimated. It is probable that if the mouth was kept normally clean and the gingiva preserved in health, diseases of the dental investment would be almost unknown. This statement is made advisably with a full realization that there are other factors to be considered, and that it is possible to tolerate filth in the mouth throughout life without the development of periodontoclasia. If the gingiva can be maintained in health, despite the neglect of mouth hygiene, under certain conditions, periodontoclasia will not develop; the real problem is the maintenance of the gingiva in health under conditions so adverse.

Another of the sources of gingival irritation is faulty restorative operations, as represented by banded and shell crowns, partial dentures, overhanging edges of fillings, defective approximal contact points, etc. All are familiar with this class of gingival irritation, which needs only to be mentioned to be recognized. Ignorance or carelessness is its explanation. Education of those responsible for such conditions is the only remedy. This must be done with the undergraduate and be emphasized as it has not been in the past.

A third source of gingival irritation is trauma, by which is meant some injurious agent introduced from without, and producing an injury such as would result from the incorrect use of the tooth-brush, ligatures, clamps, etc., and from the impaction of food between teeth with defective contact points.

This briefly summarizes the chief causes of gingival irritation, which latter is regarded as being, in many instances at least, a precursor of periodontoclasia. Black says on this point: "Some form of gingivitis always precedes the formation of a pus pocket." On the other hand, Hopewell-Smith says: "Pyorrhea alveolaris does not begin as a gingivitis. It seems impossible to imagine that if the gingivitis was, alone or combined with tartar, responsible for the pathological conditions, there could be no alteration in the bone *at the apices of the roots*, or in *situation far removed* from the surface of the gum. But this occurs."

In this Hopewell-Smith is probably in error. The changes which he observes to have taken place "at the apices of the roots, or in situations far removed from the surface of the gum," may not, and probably were not, directly caused by gingivitis, but were the changes which are known to take place as a result of that form of irritation conveyed to the entire periodontal tissues, known as traumatic occlusion, and which may exist and cause the changes referred to above wholly independent of gingivitis.

### TRAUMATIC OCCLUSION

Chief among those mechanical irritants which are of such a nature as to cause injury to the entire pericementum, and if long enough continued, to the alveolar process also, is traumatic occlusion. As one of the causes of periodontoclasia, this is a form of irritation difficult to define. It would seem to be due primarily to a lack of harmonious co-ordination of the inclined planes of the teeth when brought into occlusion, in the process of mastication. The result is that the force which should be dissipated through a slight play of tooth on tooth is communicated to the supporting tissues of the teeth in such a way as to force them outside their normal limits of motion. The motion is at first very slight, but rapidly increases with the increasing mobility of the teeth and is followed by more or less marked changes in the pericementum and alveolar process. The first of these to occur is thickening of the pericementum with a coincident absorption of the edge of the alveolar process at the gingival margin. Regarding these changes on the pericementum, Hopewell-Smith says: ''Normally measuring in middle age from 0.2 to 0.3 mm., it may extend to the enormous width of 1.0 mm.'' Equally marked changes occur at the apical region, he asserts. The alveolar bone, he says, ''exhibits even before the gum the most important metamorphoses which have occurred. Briefly they are those produced by the process of halisteresis (lack of lime salts). Not only is the free margin of the bony socket absorbed by osteoclasts near the upper and lower parts of the cervical regions of the teeth, but deep down at the radicular portion, giving the surface an eroded appearance; moreover, there is also a decalcification of the most superficial portions.'' As indicated above, Hopewell-Smith does not ascribe these changes to traumatic occlusion, but to osseous atrophy. In this I believe he is mistaken.

There are several factors which contribute to this occlusal maladjustment, such as abnormal tooth forms, habits of occlusion, malocclusion, restorative defects, etc. The outstanding fact is that the occlusal surfaces of the teeth show very little wear; the cusps are generally long, limiting thereby the triturating motion of tooth against tooth. If a study be made of ancient skulls, quite a different condition will be found to prevail; the occlusal surfaces of the teeth will show marked wear as a result of mastication, often to the extent of complete obliteration of cusps. It will also be noted that the bony investment of the teeth is intact and usually of considerable thickness, showing that the mastication to which these teeth were subjected, while great enough to completely obliterate the cusps, in no way impaired the health of their investment. The very fact that the cusps are so worn may be its own explanation. The coarse food used required great force in mastication, but the teeth were saved from the wedging effects of such force by the wear of their occlusal surfaces, permitting greater freedom of motion of tooth against tooth.

Another factor to be considered in the study of traumatic occlusion is the thickness of the alveolar process. As is known to every dentist, this varies greatly with individuals and races. Teeth supported by a thin osseous investment will not withstand the strain that teeth will more firmly supported; when subjected to adverse conditions they are more easily disturbed. The use of soft food requiring little mastication has resulted in small and undeveloped jaws, with teeth the roots of which are subnormal in size and supported by an alveolar process much thinner than that of primitive man.

These, briefly, are some of the conditions which make for and explain that form of irritation affecting the entire periodental tissues known as traumatic occlusion and which unless recognized and corrected will make a cure impossible in any case.

### SECONDARY ETIOLOGICAL FACTORS

A consideration of the secondary etiological factors of periodontoclasia involves a consideration of the possible relationship of systemic disorders to this disease. This is a point around which wordy battles have been waged, and conflicting and often irreconcilable theories have been advanced. There are those who believe that periodontoclasia in its various manifestations is but an expression of some more or less serious systemic disorder, some going so far as to assert that certain diseases, such as diabetes, tuberculosis, syphilis, etc., express themselves in the mouth in characteristic types of periodontoclasia.

An example of the wide diversity of opinion on this point held by leaders of the profession, the following is cited: Hopewell-Smith, in his recent book on "Normal and Pathological Histology of the Mouth," says regarding what he terms "pyorrhea; etiology, undetermined at present, but probably constitutional diseases coupled with infection of the gum margins with pyogenic cocci, may briefly be considered potent factors in its causation. Pyorrhea alveolaris does not initiate, but is produced by the same septic causes which lead to general systemic affections."

Black, in his book on "Special Dental Pathology," says: "It must be recognized that the general physical condition of an individual will have its effects on the progress of such a lesion in the mouth, yet I can not today, from a review of the literature, or from my own investigations, believe that more than a very limited number of cases originate from systemic causes. I have gradually come more and more to the belief that careful observation and records during the early stages will establish the fact that there is a local exciting cause in nearly every case."

Periodontists everywhere are more and more inclining to the latter belief. While recognizing the possible effects of constitutional disorders upon the etiology and progress of periodontoclasia, they have regarded such conditions in the light of possible predisposing rather than exciting causes. For convenience sake they have classified these secondary factors as abnormal systemic conditions, recognizing under that classification the possible influence upon periodontal diseases of such factors as blood supply, nutrition, faulty elimination, etc.; chemical irritations, such as might be expected to result from bacterial toxins, organic and inorganic acids, decomposition of food debris, etc.; and, lastly, predisposing local conditions, such as might obtain through structural defects in the subgingival space (which, by the way, are very common), mouth breathing, frail bony support of the teeth, etc.

As indicated above, these conditions are looked upon by the periodontist, whose large clinical experience in the treatment of these cases especially qualifies him for judgment, simply as predisposing factors which are to be taken into consideration, but which are not probably large factors, and in the absence of local exciting causes, usually nonoperative. It is not unlikely that given such predisposing factors the disease, once established by localized exciting causes, may make more rapid progress, or may not as readily yield to treatment.

Briefly summarizing the etiological factors of periodontoclasia, it may be said that they are arbitrarily divisible into two groups—primary or exciting and secondary or predisposing; that the one characteristic common to all the primary factors is that they are irritative in their nature, and depend in large measure upon this quality for the initiation of periodontal diseases; that this irritative feature may express itself at the gingival margin only, where it brings about a solution of continuity in the floor of the subgingival space, or throughout the entire supporting tissues of the teeth, each manifesting itself in a pathology wholly characteristic, yet entirely unlike the other; that the secondary or predisposing factors, as their classification indicates, are dependent for the exercise of their influence upon local exciting causes; that the one outstanding fact in the prevention of periodontal lesions is the maintenance of the gingiva in health; and that the chief instrument to that end is mouth hygiene, than which nothing is of more importance in the whole realm of dental practice.—*Journal National Association* for March.

